# Methodology for computed tomography characterization of commercially available 3D printing materials for use in radiology/radiation oncology

**DOI:** 10.1002/acm2.13999

**Published:** 2023-04-24

**Authors:** Madison Kozee, Joseph Weygand, Jacqueline M. Andreozzi, Dylan Hunt, Bradford A. Perez, Jasmine A. Graham, Gage Redler

**Affiliations:** ^1^ Department of Radiation Oncology Moffitt Cancer Center Tampa Florida USA

**Keywords:** 3D printing, additive manufacturing, material characterization, phantom

## Abstract

3D printing in medical physics provides opportunities for creating patient‐specific treatment devices and in‐house fabrication of imaging/dosimetry phantoms. This study characterizes several commercial fused deposition 3D printing materials with some containing nonstandard compositions. It is important to explore their similarities to human tissues and other materials encountered in patients. Uniform cylinders with infill from 50 to 100% at six evenly distributed intervals were printed using 13 different filaments. A novel approach rotating infill angle 10^o^ between each layer avoids unwanted patterns. Five materials contained high‐Z/metallic components. A clinical CT scanner with a range of tube potentials (70, 80, 100, 120, 140 kVp) was used. Density and average Hounsfield unit (HU) were measured. A commercial GAMMEX phantom mimicking various human tissues provides a comparison. Utility of the lookup tables produced is demonstrated. A methodology for calibrating print materials/parameters for a desired HU is presented. Density and HU were determined for all materials as a function of tube voltage (kVp) and infill percentage. The range of HU (−732.0–10047.4 HU) and physical densities (0.36–3.52 g/cm^3^) encompassed most tissues/materials encountered in radiology/radiotherapy applications with many overlapping those of human tissues. Printing filaments doped with high‐Z materials demonstrated increased attenuation due to the photoelectric effect with decreased kVp, as found in certain endogenous materials (e.g., bone). HU was faithfully reproduced (within one standard deviation) in a 3D‐printed mimic of a commercial anthropomorphic phantom section. Characterization of commercially available 3D print materials facilitates custom object fabrication for use in radiology and radiation oncology, including human tissue and common exogenous implant mimics. This allows for cost reduction and increased flexibility to fabricate novel phantoms or patient‐specific devices imaging and dosimetry purposes. A formalism for calibrating to specific CT scanner, printer, and filament type/batch is presented. Utility is demonstrated by printing a commercial anthropomorphic phantom copy.

## 1 INTRODUCTION

Additive manufacturing, or three‐dimensional (3D) printing, allows for application‐specific designs to be developed inexpensively in‐house as an alternative to ordering commercially available items from a manufacturer. The applications of this method are constantly expanding as 3D printers become more accessible. 3D printing applications are used across many scientific and medical fields. In particular, this technique is being studied for applications in radiology and radiation oncology.^1^, ^2^ There are multiple applications of additive manufacturing to be used in radiation therapy including compensators to affect beam intensity, shielding to protect organs at risk, and medical phantoms.^2^, ^3^

Fused deposition manufacturing (FDM) is a 3D printing technique that uses a spool of material to create a 3D object layer by layer. There are many materials available to be used that may be compatible with a single 3D printer, which allows for in‐house creations to accommodate diverse characteristic needs with careful choice of printing parameters and filament material. Additive manufacturing has already been implemented in radiology and radiation oncology, however, it is not yet standardized nor ubiquitous. Phantoms are an important application of additive manufacturing in radiology and radiation oncology. In addition to shape and size variations, creating phantoms using 3D printing can simulate patient inhomogeneity allowing for more accurate anthropomorphic dosimetry phantoms used in designing treatments. For example, with additive manufacturing tissue‐ and bone‐equivalent material have been used in a single phantom.^4^ Moreover, phantoms have been created using a combination of 3D printing and traditional subtractive manufacturing.^5^ The combination of materials within a phantom also expands the options for mimicking areas of patients in a more accurate way for use in image calibration and dosimetry. In addition to phantoms, additive manufacturing can improve many other aspects of radiotherapy. 3D‐printed forms for shielding have shown to be useful in protecting organs at risk in both patient‐specific ways as well as generic prints that can be made as needed in‐house.^3^

Various studies in the literature have investigated using 3D printing for the production of radiology and/or radiation oncology phantoms that mimic the radiographic properties of human tissues. The capability to produce anthropomorphic shapes has been useful in dosimetric studies.^6^ Additionally, printing techniques and parameters can be controlled to reproduce desired density and radiologic characteristics. The most common parameter to vary has been infill percentage (the percent of the inner component of a 3D print that is filled with material versus air) and the infill pattern.^7^, ^8^, ^9^, ^10^, ^11^, ^12^, ^13^, ^14^ Infill printing pattern has also been varied to avoid or control coherent textures in printed components; however, most previously published techniques use repeating patterns that are visible in radiographs/computed tomography (CT) and affect radiation dosimetry, both in ways that are not representative of human tissues.^7^, ^11^, ^13^, ^14^ The other option to produce variable density/physical properties is to use filament materials of differing composition. Certain common FDM printing plastics have been custom doped with higher atomic number (Z) materials (e.g., ABS with added barium sulfate^8^ or bismuth).^15^ There are also commercially available materials with more complex material mixtures providing varying composition that have been investigated to mimic soft tissues.^9^, ^16^, ^17^, ^18^ It has proven more challenging to print components adequately mimicking properties of human bone with higher Z composition as well as variable densities between cancellous and cortical bone. Recent studies have achieved this by incorporating commercially available materials consisting of plastics (e.g., PLA, ABS) doped with higher Z materials.^11^, ^12^, ^13^, ^14^ Some studies have also investigated the use of dual‐nozzle printers to simultaneously print multiple materials within a 3D component to control average density/radiologic properties within small subvolumes.^12^, ^13^, ^19^

The growing literature on this subject will help to find more applications and wider utility for 3D printing in radiology and radiation oncology. However, there is wide variability in printing techniques, measurement approaches, filament compositions (by manufacturer and by specific filament batch), as well as how printed components will be used (radiography/mammography,^10^, ^14^, ^16^, ^17^, ^18^, ^20^, ^21^ kilovoltage CT of various energies,^8^, ^9^, ^11^, ^12^, ^15^, ^19^, ^21^, ^22^ megavoltage applications such as MVCT or radiotherapy dosimetry,^6^, ^7^, ^12^, ^13^ or for exogenous components (e.g., bolus, orthopedic implants, brachytherapy applicators, etc.).^2^, ^3^ This work seeks to comprehensively evaluate commercially available 3D printing filaments with nonstandard composition for use in reproducing Hounsfield units (HUs) at various peak kilovoltage (kVp) spanning the range of all expected human tissues (from lung to dense bone) as well as even higher‐density/higher‐Z metallic implants. 3D‐printed cylindrical plugs of various materials/densities are compared with known and commonly used manufactured plugs. This will provide a lookup table for those seeking to 3D print anthropomorphic phantoms. However, recognizing the many variables mentioned above that could alter results for each printed component, this work presents a formalism for calibration of these printed materials under given conditions and circumstances to fine‐tune the starting point provided by the lookup tables herein. Additionally, to eliminate issues with coherent patterns, a simple approach to modifying the infill deposition geometry is provided.

## 2 METHODS AND MATERIALS

### 2.1 Filament materials and print parameters

**FIGURE 1:**
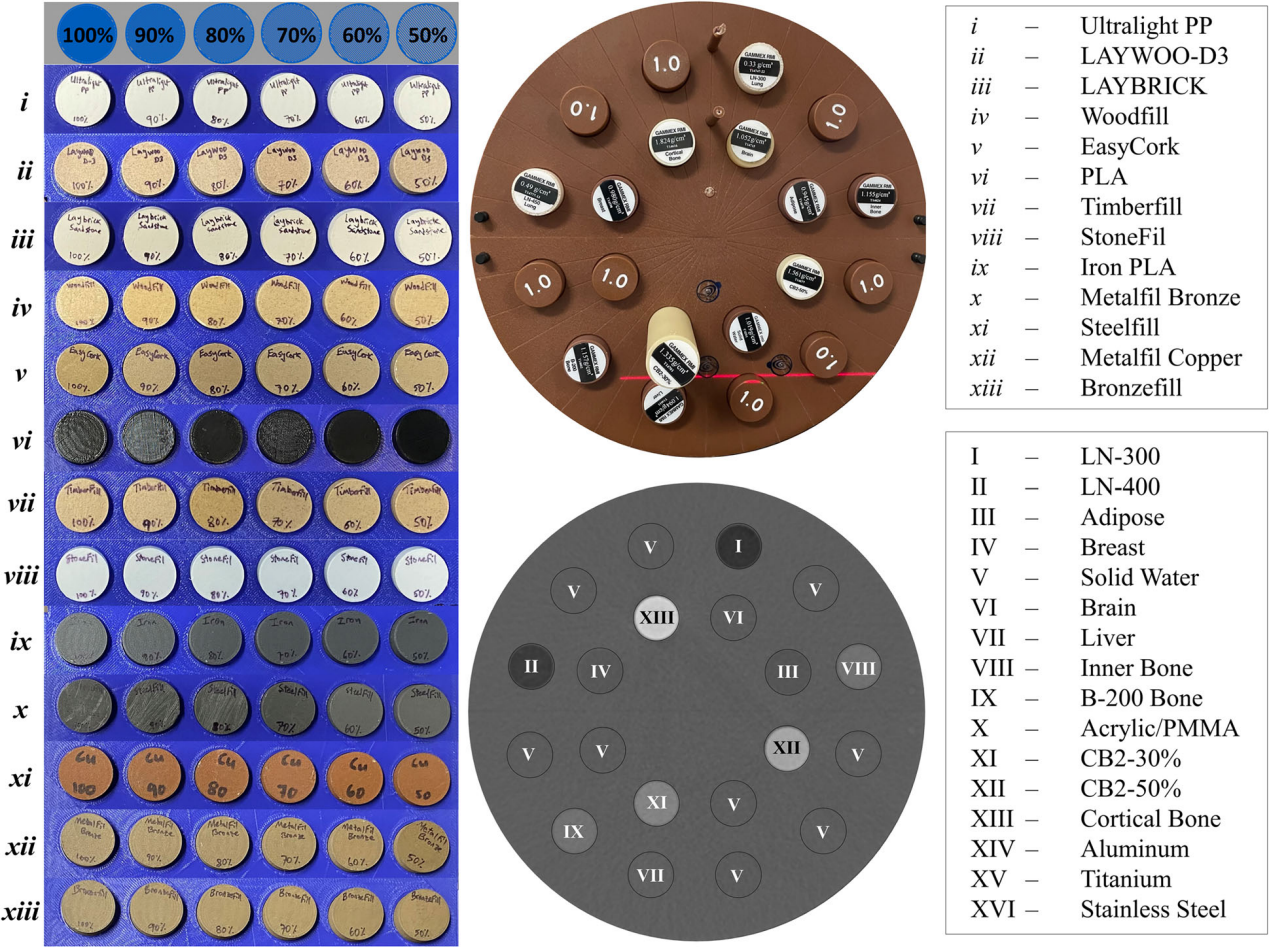
(Left) Compilation of all 3D‐printed cylindrical plugs of varying materials (see the legend on the right) with infill density varying from 50 to 100%. (Middle) The top shows the standard Gammex phantom with CT calibration plugs of different physical properties and the bottom shows the variable HU in a CT scan of this phantom with plugs labeled based on the legend to the right. Titanium(XV; *ρ* = 4.5 g/cm3) and stainless steel(XVI; *ρ* = 8.0 g/cm3) are not pictured.

The 13 different commercially available filaments used in this work are listed in Table 1, including the name, manufacturer, quoted versus measured physical density, and relevant details on the composition. Aside from PLA, these were selected due to their more complex mixed material compositions. For standardization, the radiographic properties of these printed materials are compared to the widely used Gammex phantom components (Gammex RMI, Middleton, WI, USA). In addition to varying filament composition, the physical density of a printed component was controlled using variable infill density (50%–100%, in 10% increments). Figure 1 shows the 3D‐printed cylindrical plugs for all thirteen materials and six infill settings as well as the Gammex phantom and plugs with associated CT. The roman numeral legend in Table 1 and Figure 1 will be used throughout.

**TABLE 1:** List of commercially available filaments evaluated in this work.

ID	Name	Manufacturer	Quoted filament density [g/cc]	Measured filament density [g/cc]	Composition details
***i***	Pegasus PP‐HGS25 Ultralight	FormFutura	0.75	0.65	Polypropylene (PP) filament enhanced with hollow glass nanotechnology made from soda‐lime borosilicate glass.
***ii***	LAYWOO‐D3	CC‐Products	–	0.89	Proprietary mixture containing up to 40% recycled wood fibers.
***iii***	LAYBRICK Sandstone	CC‐Products	–	1.19	Proprietary mixture containing mineral (superfine milled chalk/sandstone) components.
***iv***	Woodfill	ColorFabb	1.15	1.17	Proprietary mixture containing recycled wood fibers.
***v***	EasyCork	FormFutura	1.03	1.19	PLA‐based gravimetrically filled with 30% cork fibers.
***vi***	Poly‐Lactic Acid (PLA)	MakerGear	1.24	1.27	
***vii***	Timberfill	Fillamentum	1.25	1.21	Wood‐based biodegradable material.
***viii***	StoneFil	FormFutura	1.70	1.56	PLA‐based gravimetrically filled with 50% powdered stone.
***ix***	Magnetic Iron PLA	Protoplant	1.85	1.90	PLA‐based with iron powder additive; ferromagnetic.
***x***	Metalfil Bronze	FormFutura	3.50	3.17	PLA‐based gravimetrically filled with 80% bronze.
***xi***	Steelfill	ColorFabb	3.13	3.10	PLA‐based with 25%–30% stainless steel particle fill.
***xii***	Metalfil Copper	FormFutura	3.40	3.58	PLA‐based gravimetrically filled with 80% copper.
***xiii***	Bronzefill	ColorFabb	3.90	3.56	PLA‐based with 40% bronze powder fill.

**FIGURE 2:**
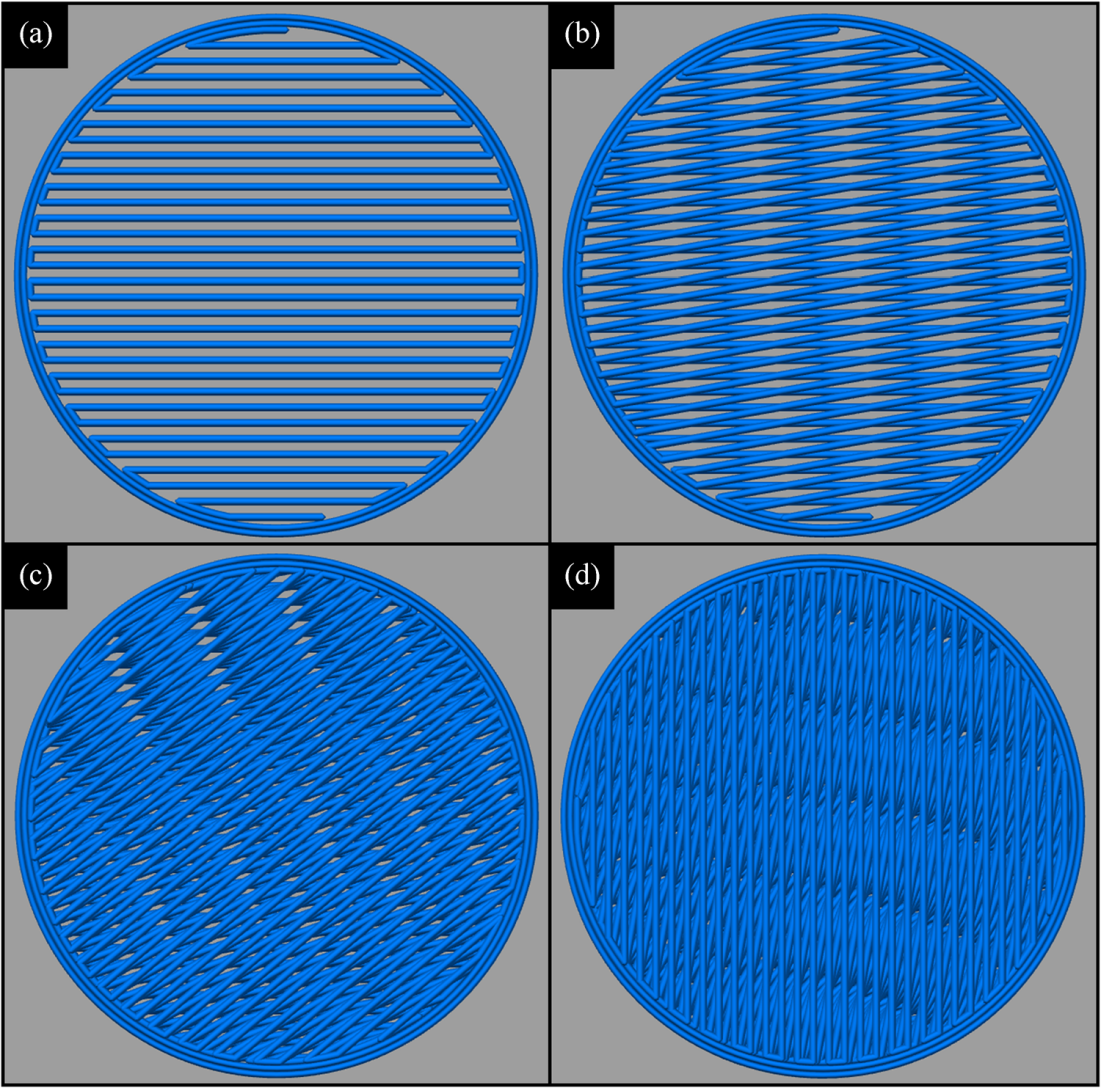
Depiction of the 10^o^ infill angle rotation with subsequent layers to approximate uniformity in printed objects. (a) Single 0.2 mm layer with 50% infill, (b) two layers or 0.4 mm, (c) five layers or 1.0 mm, (d) ten layers or 2.0 mm.

The printer used for this work is the MakerGear M3‐ID (MakerGear, LLC, Beachwood, OH, USA) with a 3.5‐mm brass nozzle. A 0.2 mm layer height was used with a 230°C extrusion temperature, printing speed of 2500 mm/min, and heated bed temperature of 50−70°C. Other print/slicer settings were extrusion multiplier of 1.0, retraction distance of 2.0 mm, retraction speed of 6000.0 mm/min, first layer speed of 20%, three bottom solid layers, two outline shells, infill extrusion width of 120%, solid infill underspeed of 80%, *x*/*y* axis speed of 18000 mm/min, *z* axis speed of 1200 mm/min. Filament diameters were all 1.75 mm. Common standard infill setting involves alternating layer perpendicular cross‐hatching (+45^o^/−45^o^), with other repeating patterns investigated in the literature (e.g., hexagonal,^11^, ^12^, ^15^ nested bricks,^9^ zig‐zags,^9^, ^11^ triangles,^11^ gyroids^13^). However, periodicity layer to layer is often seen to result in unwanted structure that will alter radiation interactions with the components, manifesting in unrealistic image characteristics and inaccurate dosimetric properties. This was seen in previous work using 3D printing to generate radiation beam attenuating compensators, where the repeating lattice structure resulted in vast differences in attenuation across a solid 3D‐printed component due to “tunnels” effectively being generated where cross hatches did not overlap.^23^ To avoid this and coherent structure visible to high‐resolution CT, the infill angle is varied by 10^o^ with each layer (nominal angular setting within slicer software conversion from 3D model to printer gcode). This creates a spiraling effect that spreads out the air component for any infill less than 100% and approximates uniformity (See Figure 2).

### 2.2 Density and HU measurements

The physical properties of all 13 filament materials were measured. Each filament had a consistent diameter of 1.75 mm (most consistently available diameter for the various materials/manufacturers and known to be compatible with the printer/nozzle/settings used in this work). The preprint filament material density was measured (see Table 1) as well as the density of the printed cylinders. Physical density measurement was based on using a 0.5‐mg precision scale (Voyager Pro Analytical Balance—VP214DCN, Ohaus Corporation, Pine Brook, NJ) to determine mass, which was then divided by volume. For the preprint filaments, a piece (>30 cm) was cut from the spool, and volume was calculated as length × π × (diameter/2)^2, with length measured to within ± 0.5 mm and diameter assumed to be to manufacturer specification (1.75 mm). For 3D‐printed cylinders, volume was calculated as height × π × (diameter/2)^2, with height and diameter equal to 2 cm. Some deviation in density was noted between manufacturer specifications and measured values. The reduction of infill caused linearly decreasing mass in the cylinders of equivalent print volume (i.e., linearly decreasing density for a given component).

The materials used were separated into two categories, higher‐ and lower‐density/Z filaments. These separate categories generally represent endogenous tissues (lower‐density/‐Z: *i–viii*) versus exogenous metallic material (higher‐density/‐Z: *ix–xiii*). The known standard materials from Gammex were similarly sorted (lower‐density/‐Z: I–XIII vs. higher‐density/‐Z: XII–XV). These materials were printed with the above‐detailed parameters and variable infill settings into cylinders with 2 cm height and 2 cm diameter.

A clinical CT scanner (Siemens Somatom Confidence CT scanner, Siemens Medical Solutions USA, Inc., Malvern, PA, USA) was used to image and measure HU for each of the 13 3D‐printed materials with six different infill percentages (78 total cylinders) across five separate kVp energy levels (70, 80, 100, 120, and 140 kVp). For the higher‐density/higher‐Z materials, the extended HU range was enabled (retains image bit depth but uses 10 rather than 1 HU spacing). At higher HU values, the measurements became unstable in highly attenuating materials causing saturation as values approached 13 000 HU. The materials found to be too attenuating to generate meaningful signal in projection data are excluded (plots truncated at 12 500 HU) as they meet saturation to avoid inaccuracies in trendlines. The commercial phantom with GAMMEX inserts that mimicked radiological properties of various human tissues, bones, and water was also imaged and HU measured at each energy level. This proved useful in determining accurate calibration, comparing the measured HU of the water equivalent insert, and it allowed the range of HU values compared to energy levels to be determined and compared to the 3D‐printed materials.

### 2.3 Data analysis

Images acquired by the CT scanner were analyzed using Mirada RTx (Mirada Medical, Oxford, UK). An interior cylindrical region with a volume of 0.79 cm^3^ (1‐cm height, 1‐cm diameter) centered within each 3D‐printed cylinder was used to find an average HU value. These average HU values were plotted, along with similar values for the standard commercial phantom materials, as a function of filament material, infill percentage, and CT kVp.

### 2.4 Anthropomorphic phantom validation

The final end‐to‐end validation of this approach is represented in the 3D print reproduction of a CT slice from a commercial thorax phantom (Computerized Imaging Reference Systems (CIRS), Inc., Norfolk, VA, USA). The CT scan of the commercial anthropomorphic phantom was imported into Raystation treatment planning software (RaySearch Laboratories AB, Stockholm, Sweden) and different tissue types (lung, soft tissue, sternum cartilage, rib, inner spine (vertebra cancellous bone), outer spine (vertebra cortical bone)) were segmented. The DICOM RT structure file was then manipulated in the open‐source 3D Slicer software^24^ to generate stl files for conversion to gcode printer files using Simplify3D (Simplify3D, Cincinnati, Ohio, USA). Prior to conversion, margins were used to ensure proper fit when piecing the components together. Lungs, sternum cartilage, and inner spine were contracted by 0.5 mm isotropically. Ribs were also contracted by 0.5 mm unless they abutted lung (to avoid gaps where both would be contracted). The portion of the soft tissue containing the outer spine was contracted by 0.5 mm to allow a snug fit. This approach is generally similar to that used by Tino et al. previously.^13^

Material and infill print settings were selected based on the lookup table generated from printed cylinder measurements to best correspond to the desired HU found for different components of the commercial anthropomorphic phantom. Material known to contain calcium (StoneFil, viii) was selected to print bone components. Data from printed cylindrical plugs were interpolated (or, in the case of low‐density lung, extrapolated) to more closely match HU values. Each separate material component was printed separately and pieced together to construct the full slice from the anthropomorphic phantom. Due to printer bed size limitations, the larger general soft tissue component was printed in four separate quadrants. The CT of the 3D‐printed mimic was acquired at 120 kVp with 1 mm slices.

## 3 RESULTS

**FIGURE 3:**
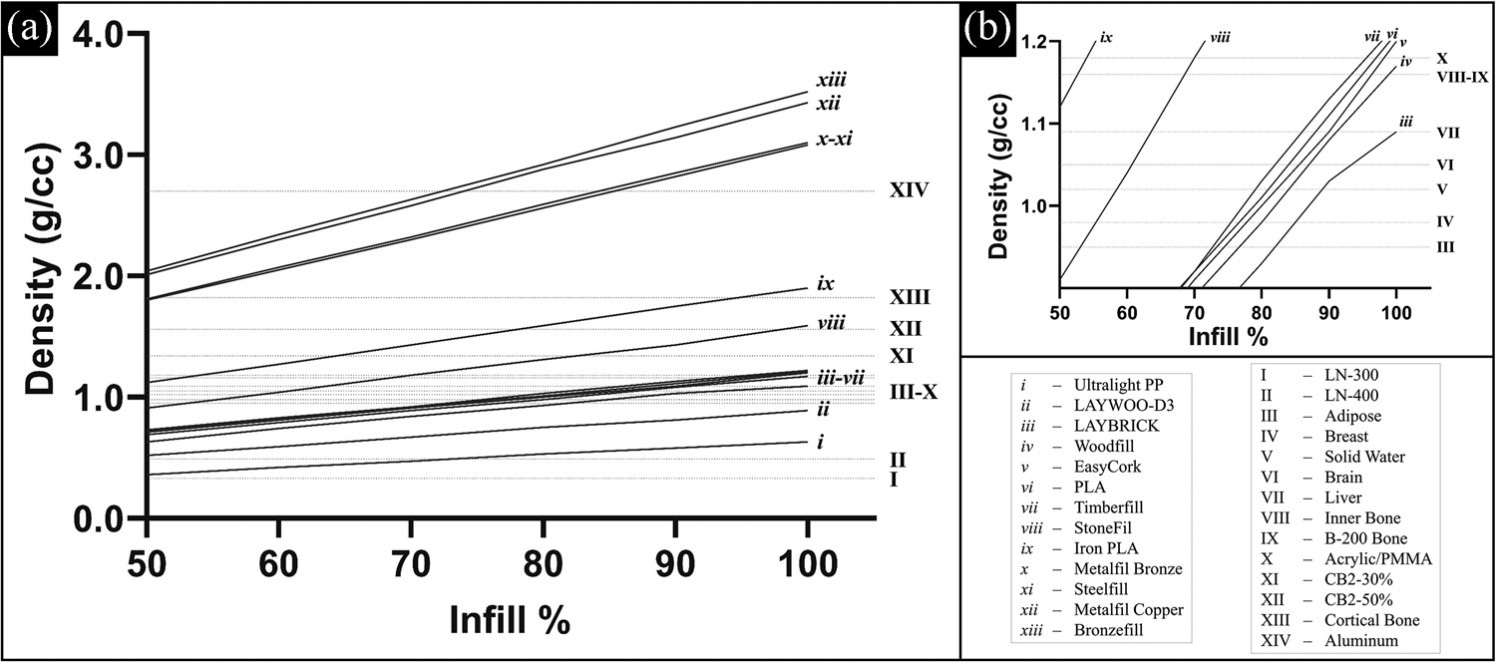
(a) Physical density versus infill percentage for all tested materials. Dashed horizontal lines show standard commercial phantom materials representing human tissue for comparison. (b) Zoomed in portion to more easily visualize the more closely packed values between 0.9 and 1.2 g/cc.

The physical densities measured for the different cylinders printed with the various materials and infill percentage settings are shown in Figure 3. As expected, physical density for the 3D‐printed cylinders varied linearly with the infill percentage. Physical densities of standard commercial phantom materials representing human tissues are also plotted to show the intersection of multiple choices of 3D print material and infill setting with these standards. Figure 3b shows a zoomed‐in portion of the plot to better visualize the more clustered data points for materials that were predominantly plastic (PLA) and had densities in the 0.9–1.2 g/cc range.

**FIGURE 4:**
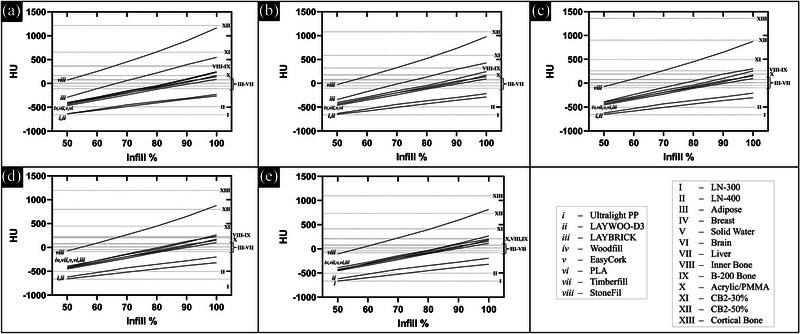
HU versus infill percentage for lower‐density/‐Z materials (i–viii) plotted for CT scans with (a) 70 kVp, (b) 80 kVp, (c) 100 kVp, (d) 120 kVp, and (e) 140 kVp. Overlap with all standard commercial phantom materials representing human tissue (I–XII) aside from cortical bone (XIII) can be seen.

**FIGURE 5:**
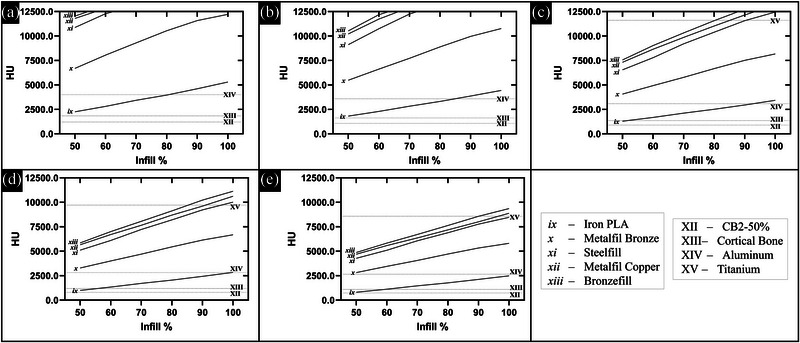
HU versus infill percentage for higher‐density/‐Z materials (ix–xiii) plotted for CT scans with (a) 70 kVp, (b) 80 kVp, (c) 100 kVp, (d) 120 kVp, and (e) 140 kVp. Overlap with standard commercial phantom materials representing more dense bone (XII–XIII) and exogenous aluminum (XIV) and titanium (XV) can be seen. Insufficient signal in CT projection data prevented evaluation of overlap with stainless steel (XVI) as this would fall beyond the stable HU range (truncated at 12 500 HU). HU, Hounsfield unit.

The CT scans using several energy levels and various materials and densities of each led to a spectrum of trendlines that overlapped at multiple points with many of the phantom HU values. This shows that, with varying infill and energy levels, the same radiological measurements can be made with various 3D printing materials corresponding to those of the standard commercial phantom materials representing various human tissues. Figure 4 shows these data for the lower‐density/‐Z group of materials and Figure 5 shows these data for the higher‐density/‐Z group of materials (raw data in Tables S2 and S3). The ordinate in Figure 5 is truncated at 12 500 HU since the extended HU range values were found to become unstable beyond this range (due to lack of signal in projection data for image reconstruction). Stainless steel is more attenuating and would fall beyond this range (and, therefore, is not in the plots). Similarly, Steelfill (xi), Metalfil Copper (xii), and Bronzefill (xiii) become too attenuating to produce stable HU values. Error bars for the HU measurements are not included in the plots for Figures 4 and 5 to avoid obscuring trendlines but the standard deviation of the HU within each cylinder was on average 16.9 (±2.0). Furthermore, using this HU standard deviation in a given cylinder as a measure of uniformity, the printed uniformity was found to be independent of infill percentage (linear fit of HU standard deviation versus infill percentage (%) for each material produced an average slope of 0.01 HU/%).

**FIGURE 6:**
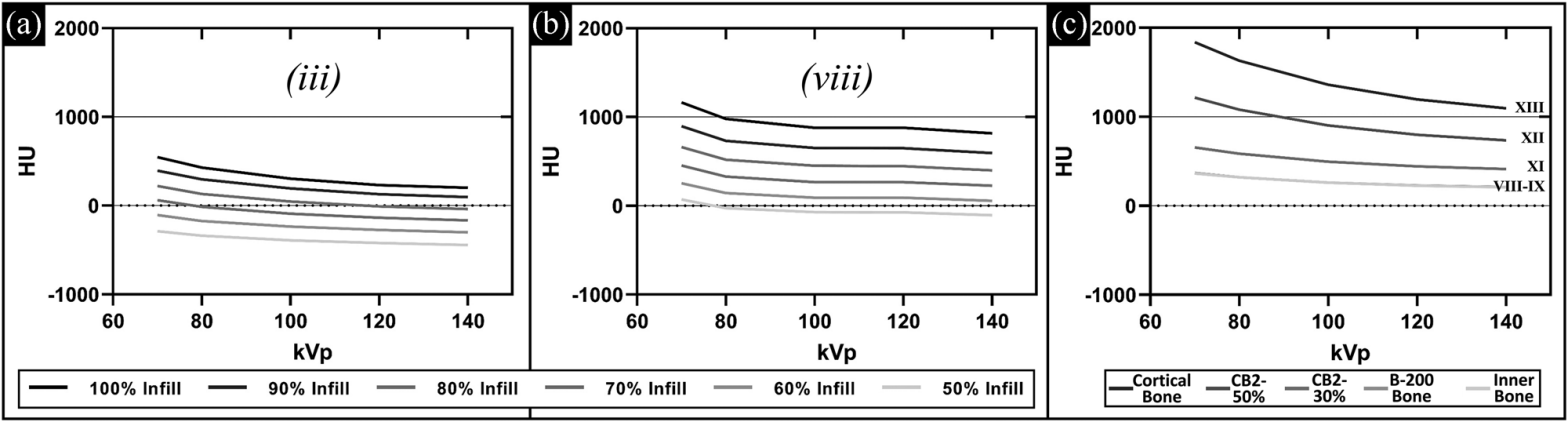
HU versus kVp for different infill settings in filaments containing calcium: (a) LAYBRICK (iii) and (b) Stonefil (viii). Similar plots for the bone mimicking standard phantom materials are shown in (c). HU, Hounsfield unit.

Figure 6 shows the dependence of HU values on kVp for the two lower density 3D print filaments known to contain calcium (LAYBRICK(iii) and StoneFil(viii)) and, therefore, more accurately mimic the physical and radiologic properties of bone tissues. Figure 6c shows similar HU versus kVp plots for bone mimicking standard commercial phantom materials. This shows similar behavior with increased HU due to increased beam attenuation via photoelectric effect ($\propto \frac{Z^{3}}{E^{3}}$) in the higher‐Z calcium components at lower energy between the 3D print materials and the standard phantom materials.^25^ This demonstrates that these filaments can be selected appropriately to both reproduce physical densities (more important for Compton interactions) for various bone types (e.g., cancellous or cortical) as well as the atomic number (more important for photoelectric interactions).

**FIGURE 7:**
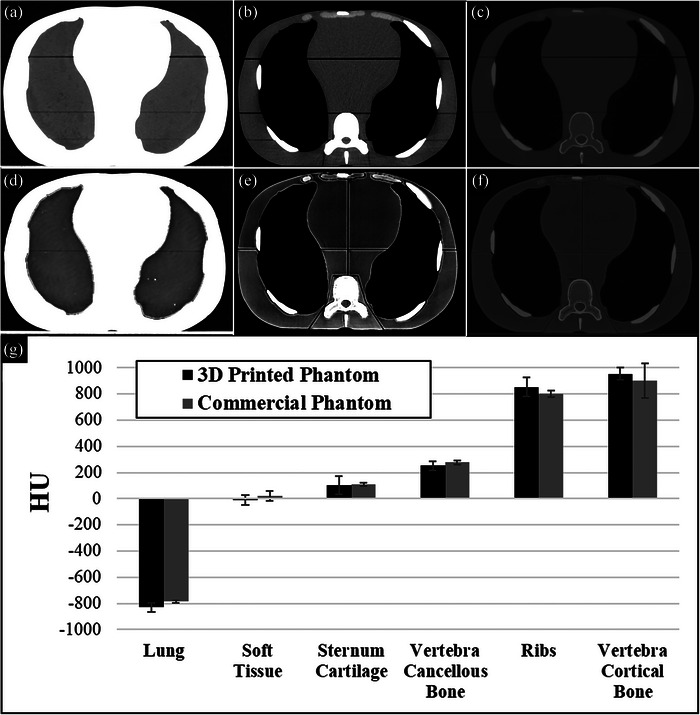
CT scan of commercial anthropomorphic phantom with varying window (W) and level (L) to visualize relevant tissue types shown in (a) lung (*W* = 500 HU, *L* = −800 HU), (b) thorax (*W* = 350 HU, *L* = 40 HU), and (c) bone (*W* = 3765 HU, *L* = 730 HU). The same is shown for the 3D‐printed version in (d)–(f), respectively. (g) Quantitative comparison of an average HU in each tissue type printed versus that in the commercial phantom shows all values overlap within one standard deviation (error bars). HU, Hounsfield unit.

Validation that the data and method of printing and CT scanning plugs with a given filament and variable infill percentage presented herein can be used to calibrate how to use 3D printing to reproduce desired HU is given by 3D printing a corresponding CT slice from a commercial anthropomorphic phantom with matching HU. To this end, Figure 7 shows the results of applying this method. The images of the commercial phantom are in Figure 7a–c (varying HU window (W) and level (L) to visualize relevant tissue types: lung (*W* = 500, *L* = −800), thorax (*W* = 350, *L* = 40), and bone (*W* = 3765, *L* = 730), respectively). The different segmented tissue types in the CT (acquired at 120 kVp) of the commercial anthropomorphic phantom displayed the HU values (mean ± standard deviation) in Table 2. Based on these values, the calibration data acquired for the 3D‐printed cylinders were used to select 3D print filament and infill percentage setting (see Table 2). Using these materials/infill percentages, the corresponding values for the CT HU of the 3D‐printed phantom (also acquired at 120 kVp) are given in Table 2. The CT scan of the 3D‐printed version with the same lung, thorax, and bone window/level is shown in Figure 7d–f, respectively, for qualitative comparison. Figure 7g quantitatively shows that average HU values within the various regions (printed with different materials/infill percentage) are relatively well reproduced with 3D printing (error bars representing one standard deviation are overlapping). Note that in general, the error bars are larger for the 3D‐printed phantom versus the commercially produced phantom. This is not the case for the vertebra cortical bone due to the introduction of uncertainties associated with contour precision and volume averaging in CT voxels for this thin outer portion of the vertebra confounding uncertainty due to material variability alone. Horizontal lines (air) in the commercial phantom images (Figure 6a–c) are gaps in separate stacked components designed for the placement of dosimetric film. Similar lines (splitting phantom into quadrants and surrounding the spine region) in the 3D‐printed phantom are air gaps where components were pieced together.

**TABLE 2:** Comparison of HU values for 120‐kVp scan of anthropomorphic phantom and corresponding 3D‐printed phantom materials

Tissue type	Commercial phantom HU	3D‐printed phantom HU	3D print filament	3D print infill %
Lung	−785.7 ± 11.5	829.6 ± 33.7	*i*	25%
Soft tissue	22.3 ± 38.6	−9.5 ± 37.4	*vi*	84%
Sternum cartilage	107.1 ± 11.7	102.8 ± 69.2	*v*	95%
Vertebra cancellous bone	276.8 ± 15.3	253.1 ± 34.4	*viii*	70%
Ribs	799.0 ± 25.7	851.5 ± 72.0	*viii*	95%
Vertebra cortical bone	901.3 ± 132.0	953.9 ± 47.0	*viii*	100%

## 4 DISCUSSION

In the past decade, 3D printing capabilities have expanded. The plethora of material types commercially available and advanced printing techniques have opened exciting new applications for printing in medicine. In radiology and radiation oncology, the production of phantoms with known geometry and physical/radiologic characteristics is critical. Particularly those that can mimic human tissues and/or exogenous materials that present in patients (e.g., orthopedic implants) or aid in enhancing the personalization of radiotherapy treatments (e.g., customized bolus or brachytherapy applicators). Advancements in 3D printing have allowed for applications of in‐house additive manufacturing to grow extremely quickly in recent years. There are numerous potential applications due to the flexibility and capability of this methodology in the medical setting. Increased knowledge of material types can aid in these applications and create designs using materials that are more suitable for the need than a standard PLA filament. In radiotherapy, additive manufacturing has already been used to create compensators, phantoms, shielding, and other innovative solutions to improve standard practices.^5^, ^23^ In phantom production, the need to become more tissue equivalent is being explored and this examination of commercially available materials will allow for radiological properties to be replicated more thoroughly.^21^ The replication of tissues for use in dosimetry has been successfully used in rapid prototyping within radiotherapy.^26^ Additionally, the use of 3D‐printed patient‐specific designs can translate to improved accuracy in treatment planning and patient dosimetry.^27^ This work seeks to add to the growing body of literature characterizing 3D printing materials to facilitate increased utilization of 3D printing in these fields. In particular, this work characterized the radiologic properties of commercially available filaments containing material additives that allow the replication of a wide range of densities and atomic numbers.

One practical way to interrogate such properties is via HU values obtained from a CT scan. In addition to radiology applications, this is relevant in radiotherapy also, as CT HU is often converted to physical/electron density and/or material composition to model particle interactions and perform dose deposition calculations. This is the method of characterization in this work. Other studies have utilized alternate methods that are either more relevant to a specific application or to isolate specific variables (e.g., linear attenuation coefficient in monoenergetic beams^15^, ^16^ or applying the Beer–Lambert law to determine effective attenuation of polyenergetic beams through different thicknesses,^10^, ^17^, ^21^ apparent kerma attenuation coefficients,^14^ x‐ray spectroscopy,^18^ Monte Carlo simulations,^14^ and even radiotherapy planned dose evaluation with gafchromic film dosimetry).^13^ Megavoltage CT has also been used, which, although it is 2.5 MV rather than the standard 6−18 MV used therapeutically, more closely models interactions governing radiotherapy dose deposition.^12^ To more rigorously characterize chemical composition (e.g., effective atomic number), dual‐energy CT has also been implemented.^28^

Previous studies have characterized 3D print materials using kilovoltage CT similar to the methods in this work.^7^, ^8^, ^9^, ^11^, ^12^, ^13^, ^15^, ^16^, ^19^, ^21^ However, the work presented herein builds upon past efforts in multiple ways. This work focuses on readily and commercially available filaments, most of which have not yet been characterized. These materials are selected as they are considered more “exotic” with various dopants added to the plastic. As such, this work presents materials and printed components that span a wider range of densities and HU than most previous publications. The materials characterized in this work include mimics for lung and beyond bone into more metallic materials. Previous studies predominantly focused on soft tissues and excluded bone or metallic materials. The extremes of human tissues (lung and bone) are particularly challenging in 3D printing. Bone is higher density generally but has a large range of density (from cancellous to cortical bone) and also has higher Z than most other tissues. Trying to match both density and atomic composition is challenging.

There have been clever approaches published with dual nozzle printers interleaving multiple materials to produce the desired average properties.^13^, ^19^ However, this requires more expensive dual nozzle 3D printers, which reduces the cost–benefit associated with in‐house additive manufacturing. On the other end of the spectrum, mimicking lung tissue requires very low‐density prints. Most approaches (including this work) to varying density use decreasing infill percentage to increase the air content in a solid printed component. However, most published approaches resulted in undesirable internal structures (e.g., grids or honeycombs). There have been several approaches published using gyroid structures^13^ and/or printing speed modification^22^ to more closely mimic lung tissue. Our work has aimed to simplify the process by just varying material (all commercially available and 1–5 X the cost of standard PLA) and infill percentage, while otherwise maintaining consistency in all print settings. Our approach (first used for 3D‐printed compensators^23^) of rotating the infill print direction with each layer allows for printing of the low‐density lung mimic while avoiding structures affecting imaging (e.g., see Figure 7d with lung printed as low as 25% infill scanned with 1‐mm CT slices). It should be noted that the 10^o^ angle used in this work, while successful in achieving desired uniformity and avoiding coherent patterns from infill printing in this application, is not necessarily the optimal setting. In particular, for applications imaging with thinner slices (e.g., microCT), this parameter may need to be adjusted. Furthermore, the accuracy of the actual infill print angle relative to the nominal angle was not evaluated and this factor may change based on printer used.

This work also serves to present a methodology for readers to calibrate 3D‐printed objects for their own particular set of parameters, consisting of printing cylindrical plugs with varied infill percentage that are then CT scanned to find HU to enable interpolation to achieve the desired HU. We have generated a comprehensive lookup table (see supplemental tables for tabulated data) for the selection of initial material and print settings to produce tissue equivalent components covering low‐density lung to high‐density bone. In the characterized materials, multiple materials produce the same density and HU with different settings (i.e., the cost can be saved by only using a subset of these materials). Note, there are many potential variables that could make direct reproducibility challenging. CT scanner parameters may vary (spectra for various kVp settings, reconstruction kernels, detector type, etc.), which would result in different HU for the same object/material. 3D printers and settings can also result in different printed objects. It is challenging to fully separate the contribution of each potential variable to uncertainty in this application. For instance, printing with the more abrasive materials containing metal particles can wear and degrade printer nozzle, which would cause the uncertainty stemming just from the 3D printing process affecting the density and HU of printed objects to vary over time. Ideally, the results presented herein will be of use for any FDM printer but, for the above‐stated considerations for variability and uncertainty, it is recommended that calibration of printed components with specific printer and settings be performed to increase accuracy. Additionally, there will be uncertainty between vendor filament batches as the quality control processes are unknown. All the above points argue for calibration to a user's own CT scanner, 3D printer, and filament spool using the lookup table as a starting point and following the approach presented herein. As the methodologies for creating in‐house 3D prints to suit specific needs improve, the need for generic items from a manufacturer may decrease and patient‐specific designs can be used more frequently. This reduces the wait times and costs associated with ordering from a manufacturer.

## 5 CONCLUSIONS

Thirteen different FDM 3D printing materials with various infill settings to control density were characterized via physical densities and kV CT HU values. The measured HUs were comparable with several human tissues (represented by established standard phantom materials in radiology/radiotherapy) spanning low‐density lung to high‐density bone as well as other materials found in medical applications. Infill print angle rotation per layer is introduced to remove the unwanted structure in 3D‐printed components, particularly at low infill percentages. This was validated by a 3D‐printed reproduction of a portion of a commercial anthropomorphic phantom with accurately reproduced HU. This survey of materials outlines characteristics for future reference in developing patient‐specific applications to be used during medical imaging and presents a methodology for others to calibrate their printing approaches to obtain desired characteristics with readily available commercial products and limited required resources. This contributes to the growing body of literature in this field that will hopefully continue to expand applicability and accessibility to task/patient‐specific phantoms in radiology and radiation oncology.

## AUTHOR CONTRIBUTIONS

All of the above‐listed authors contributed directly to the intellectual content of the paper including work design and acquisition of data, writing/editing the manuscript, and final approval of this version.

## CONFLICT OF INTEREST STATEMENT

The authors declare no conflicts of interest.

## Supporting information

Supporting Information

Supporting Information

Supporting Information

Supporting Information
