# Supplementary material for: Methodology for computed tomography characterization of commercially available 3D printing materials for use in radiology/radiation oncology
Source: J Appl Clin Med Phys. 2023 Apr 24;24(6):e13999. doi: 10.1002/acm2.13999 (PMC10243336; doi:10.1002/acm2.13999)
Supplement: Supplementary file 1 — Supporting Information [file ACM2-24-e13999-s002.docx]

**Supplemental Table 1.** Densities for raw filament, 3D printed cylinders, and Gammex plugs. Data plotted in Figure 3.

| **Material** |  | **Density [g/cm3]** | | | | | |  |
| --- | --- | --- | --- | --- | --- | --- | --- | --- |
|  | **Raw**  **Filament** | **100% Infill** | **90% Infill** | **80% Infill** | **70% Infill** | **60% Infill** | **50% Infill** | |
| i | 0.65 | 0.63 | 0.58 | 0.53 | 0.47 | 0.42 | 0.36 | |
| ii | 0.89 | 0.89 | 0.81 | 0.75 | 0.67 | 0.59 | 0.52 | |
| iii | 1.19 | 1.09 | 1.03 | 0.93 | 0.84 | 0.74 | 0.63 | |
| iv | 1.17 | 1.17 | 1.08 | 0.98 | 0.89 | 0.79 | 0.69 | |
| v | 1.19 | 1.20 | 1.09 | 1.00 | 0.91 | 0.81 | 0.71 | |
| vi | 1.27 | 1.21 | 1.11 | 1.01 | 0.92 | 0.83 | 0.73 | |
| vii | 1.21 | 1.22 | 1.13 | 1.03 | 0.92 | 0.82 | 0.72 | |
| viii | 1.56 | 1.59 | 1.43 | 1.31 | 1.18 | 1.04 | 0.91 | |
| ix | 1.90 | 1.90 | 1.75 | 1.59 | 1.43 | 1.27 | 1.12 | |
| x | 3.17 | 3.08 | 2.82 | 2.56 | 2.30 | 2.05 | 1.80 | |
| xi | 3.10 | 3.10 | 2.85 | 2.59 | 2.32 | 2.07 | 1.81 | |
| xii | 3.58 | 3.43 | 3.14 | 2.88 | 2.58 | 2.30 | 2.01 | |
| xiii | 3.56 | 3.52 | 3.23 | 2.92 | 2.63 | 2.34 | 2.04 | |
| I | 0.33 | | | | | | |  |
| II | 0.49 | | | | | | |  |
| III | 0.95 | | | | | | |  |
| IV | 0.98 | | | | | | |  |
| V | 1.02 | | | | | | |  |
| VI | 1.05 | | | | | | |  |
| VII | 1.09 | | | | | | |  |
| VIII | 1.16 | | | | | | |  |
| IX | 1.16 | | | | | | |  |
| X | 1.18 | | | | | | |  |
| XI | 1.34 | | | | | | |  |
| XII | 1.56 | | | | | | |  |
| XIII | 1.82 | | | | | | |  |
| XIV | 2.70 | | | | | | |  |
| XV | 4.50 | | | | | | |  |
| XVI | 8.00 | | | | | | |  |
