# Supplementary material for: Methodology for computed tomography characterization of commercially available 3D printing materials for use in radiology/radiation oncology
Source: J Appl Clin Med Phys. 2023 Apr 24;24(6):e13999. doi: 10.1002/acm2.13999 (PMC10243336; doi:10.1002/acm2.13999)
Supplement: Supplementary file 3 — Supporting Information [file ACM2-24-e13999-s003.docx]

**Supplemental Table 3.** HUs for standard commercial phantom materials plugs. Data plotted in Figures 4, 5, and 6.

| **Material** | **70kVp** | **80kVp** | **100kVp** | **120kVp** | **140kVp** |
| --- | --- | --- | --- | --- | --- |
| I | -653.2 | -658.8 | -661.1 | -664.7 | -665.8 |
| II | -489.4 | -492.1 | -502.4 | -502.4 | -502.5 |
| III | -108.7 | -103.5 | -93.9 | -86.6 | -80.4 |
| IV | -53.5 | -50.6 | -45.5 | -44.6 | -41.1 |
| V | 13.6 | 10.8 | 6.0 | 3.9 | 3.7 |
| VI | -2.1 | 9.5 | 20.3 | 24.4 | 30.7 |
| VII | 84.7 | 86.1 | 84.2 | 80.5 | 81.9 |
| VIII | 364.4 | 321.3 | 262.3 | 226.5 | 207.8 |
| IX | 367.9 | 321.8 | 262.3 | 227.5 | 210.4 |
| X | 166.7 | 177.1 | 194.2 | 204.8 | 210.6 |
| XI | 655.0 | 586.6 | 497.0 | 444.5 | 413.3 |
| XII | 1215.6 | 1080.6 | 903.1 | 798.0 | 735.2 |
| XIII | 1839.0 | 1630.1 | 1360.3 | 1195.8 | 1095.9 |
| XIV | 4002.3 | 3584.4 | 3089.5 | 2816.0 | 2645.9 |
| XV | 16623.8 | 14680.3 | 11604.9 | 9714.9 | 8576.3 |
| XVI | 18741.7 | 19741.3 | 20456.6 | 19010.3 | 17193.5 |

Note: HU values greater than 12,500 have diminished accuracy due to saturation and lack of transmission signal in projection data.
