# Supplementary material for: Methodology for computed tomography characterization of commercially available 3D printing materials for use in radiology/radiation oncology
Source: J Appl Clin Med Phys. 2023 Apr 24;24(6):e13999. doi: 10.1002/acm2.13999 (PMC10243336; doi:10.1002/acm2.13999)
Supplement: Supplementary file 4 — Supporting Information [file ACM2-24-e13999-s004.docx]

**Supplementary Data**

**Supplemental Table 1.** Densities for raw filament, 3D printed cylinders, and Gammex plugs. Data plotted in Figure 3.

| **Material** |  | **Density [g/cm3]** | | | | | |  |
| --- | --- | --- | --- | --- | --- | --- | --- | --- |
|  | **Raw**  **Filament** | **100% Infill** | **90% Infill** | **80% Infill** | **70% Infill** | **60% Infill** | **50% Infill** | |
| i | 0.65 | 0.63 | 0.58 | 0.53 | 0.47 | 0.42 | 0.36 | |
| ii | 0.89 | 0.89 | 0.81 | 0.75 | 0.67 | 0.59 | 0.52 | |
| iii | 1.19 | 1.09 | 1.03 | 0.93 | 0.84 | 0.74 | 0.63 | |
| iv | 1.17 | 1.17 | 1.08 | 0.98 | 0.89 | 0.79 | 0.69 | |
| v | 1.19 | 1.20 | 1.09 | 1.00 | 0.91 | 0.81 | 0.71 | |
| vi | 1.27 | 1.21 | 1.11 | 1.01 | 0.92 | 0.83 | 0.73 | |
| vii | 1.21 | 1.22 | 1.13 | 1.03 | 0.92 | 0.82 | 0.72 | |
| viii | 1.56 | 1.59 | 1.43 | 1.31 | 1.18 | 1.04 | 0.91 | |
| ix | 1.90 | 1.90 | 1.75 | 1.59 | 1.43 | 1.27 | 1.12 | |
| x | 3.17 | 3.08 | 2.82 | 2.56 | 2.30 | 2.05 | 1.80 | |
| xi | 3.10 | 3.10 | 2.85 | 2.59 | 2.32 | 2.07 | 1.81 | |
| xii | 3.58 | 3.43 | 3.14 | 2.88 | 2.58 | 2.30 | 2.01 | |
| xiii | 3.56 | 3.52 | 3.23 | 2.92 | 2.63 | 2.34 | 2.04 | |
| I | 0.33 | | | | | | |  |
| II | 0.49 | | | | | | |  |
| III | 0.95 | | | | | | |  |
| IV | 0.98 | | | | | | |  |
| V | 1.02 | | | | | | |  |
| VI | 1.05 | | | | | | |  |
| VII | 1.09 | | | | | | |  |
| VIII | 1.16 | | | | | | |  |
| IX | 1.16 | | | | | | |  |
| X | 1.18 | | | | | | |  |
| XI | 1.34 | | | | | | |  |
| XII | 1.56 | | | | | | |  |
| XIII | 1.82 | | | | | | |  |
| XIV | 2.70 | | | | | | |  |
| XV | 4.50 | | | | | | |  |
| XVI | 8.00 | | | | | | |  |

**Supplemental Table 2.** Tabulated HUs for 3D printed plugs. Data plotted in Figures 4, 5, and 6.

| **Material** | **Infill%** | **70 kVp** | **80 kVp** | **100 kVp** | **120 kVp** | **140 kVp** |
| --- | --- | --- | --- | --- | --- | --- |
| i | 100 | -261.6 | -286.9 | -302.7 | -319.8 | -317.9 |
|  | 90 | -328.2 | -356.1 | -364.5 | -380.6 | -379.7 |
|  | 80 | -405.1 | -427.6 | -434.2 | -448.2 | -451.7 |
|  | 70 | -482.1 | -504.0 | -507.8 | -516.7 | -523.3 |
|  | 60 | -562.1 | -579.3 | -582.2 | -588.7 | -597.7 |
|  | 50 | -635.5 | -649.8 | -653.5 | -657.2 | -665.5 |
| ii | 100 | -227.8 | -214.6 | -207.8 | -199.3 | -192.5 |
|  | 90 | -308.6 | -296.1 | -288.2 | -280.3 | -273.0 |
|  | 80 | -375.3 | -365.4 | -359.5 | -353.8 | -346.9 |
|  | 70 | -446.8 | -440.5 | -432.4 | -426.0 | -421.9 |
|  | 60 | -542.3 | -537.6 | -530.2 | -526.8 | -522.9 |
|  | 50 | -634.8 | -631.5 | -623.6 | -620.4 | -619.4 |
| iii | 100 | 546.6 | 428.2 | 303.5 | 231.8 | 201.9 |
|  | 90 | 393.8 | 295.7 | 193.7 | 127.5 | 97.2 |
|  | 80 | 222.1 | 132.3 | 46.7 | -9.0 | -39.1 |
|  | 70 | 61.4 | -13.0 | -91.4 | -137.2 | -166.3 |
|  | 60 | -106.7 | -173.4 | -235.5 | -275.0 | -300.7 |
|  | 50 | -287.9 | -339.7 | -391.1 | -420.4 | -444.4 |
| iv | 100 | 86.7 | 92.8 | 98.7 | 103.3 | 115.5 |
|  | 90 | -15.0 | -14.9 | -4.9 | -2.0 | 9.6 |
|  | 80 | -118.9 | -119.5 | -107.3 | -105.4 | -99.8 |
|  | 70 | -237.7 | -239.6 | -228.3 | -225.3 | -222.1 |
|  | 60 | -351.3 | -350.5 | -340.8 | -337.8 | -338.6 |
|  | 50 | -464.3 | -462.1 | -455.3 | -451.2 | -452.7 |
| v | 100 | 170.2 | 169.1 | 173.9 | 174.8 | 187.0 |
|  | 90 | 22.1 | 18.4 | 27.4 | 28.0 | 36.5 |
|  | 80 | -92.1 | -98.3 | -88.0 | -90.7 | -88.3 |
|  | 70 | -193.9 | -198.8 | -192.4 | -190.6 | -191.2 |
|  | 60 | -313.0 | -315.8 | -309.3 | -309.6 | -310.5 |
|  | 50 | -434.9 | -434.0 | -428.6 | -426.1 | -429.1 |
| vi | 100 | 240.7 | 247.9 | 257.6 | 263.1 | 267.1 |
|  | 90 | 88.0 | 94.6 | 102.4 | 106.8 | 110.9 |
|  | 80 | -52.9 | -47.4 | -40.4 | -36.5 | -33.1 |
|  | 70 | -161.2 | -156.5 | -149.6 | -146.2 | -142.9 |
|  | 60 | -291.4 | -286.6 | -281.8 | -279.1 | -276.3 |
|  | 50 | -404.2 | -401.0 | -395.9 | -393.4 | -391.0 |
| vii | 100 | 141.5 | 139.4 | 151.7 | 151.7 | 157.8 |
|  | 90 | 42.4 | 42.8 | 52.7 | 53.8 | 55.5 |
|  | 80 | -76.2 | -76.8 | -70.1 | -68.4 | -66.0 |
|  | 70 | -201.1 | -198.5 | -190.8 | -190.8 | -189.7 |
|  | 60 | -320.5 | -320.3 | -312.7 | -313.2 | -313.2 |
|  | 50 | -437.1 | -436.3 | -431.2 | -430.6 | -428.6 |
| viii | 100 | 1163.2 | 978.2 | 877.8 | 879.5 | 816.1 |
|  | 90 | 895.2 | 732.2 | 650.4 | 648.1 | 594.3 |
|  | 80 | 661.7 | 518.9 | 450.2 | 446.7 | 399.1 |
|  | 70 | 452.8 | 328.9 | 265.9 | 266.0 | 225.4 |
|  | 60 | 253.8 | 144.7 | 91.5 | 91.5 | 56.6 |
|  | 50 | 71.6 | -26.5 | -72.0 | -72.6 | -106.0 |
| ix | 100 | 5298.9 | 4444.3 | 3429.1 | 2840.4 | 2486.1 |
|  | 90 | 4614.5 | 3870.1 | 2955.9 | 2435.6 | 2116.8 |
|  | 80 | 3967.3 | 3319.2 | 2517.0 | 2049.0 | 1767.9 |
|  | 70 | 3432.1 | 2830.2 | 2118.8 | 1710.5 | 1463.8 |
|  | 60 | 2806.2 | 2295.8 | 1686.5 | 1335.1 | 1121.5 |
|  | 50 | 2257.9 | 1816.0 | 1296.9 | 989.8 | 806.8 |
| x | 100 | 13345.9 | 14224.2 | 13190.4 | 10623.8 | 8879.2 |
|  | 90 | 13431.2 | 14008.8 | 12154.0 | 9604.5 | 8005.5 |
|  | 80 | 13346.9 | 13571.3 | 11027.1 | 8690.0 | 7180.3 |
|  | 70 | 13373.1 | 12923.0 | 9856.4 | 7656.2 | 6357.8 |
|  | 60 | 12832.9 | 11737.9 | 8647.1 | 6705.9 | 5573.0 |
|  | 50 | 11796.9 | 10208.4 | 7313.6 | 5660.8 | 4696.6 |
| xi | 100 | 12214.8 | 10768.4 | 8171.4 | 6687.1 | 5803.2 |
|  | 90 | 11581.9 | 9968.1 | 7517.7 | 6136.3 | 5332.9 |
|  | 80 | 10534.3 | 8888.5 | 6671.2 | 5438.7 | 4711.6 |
|  | 70 | 9295.3 | 7723.7 | 5773.3 | 4699.8 | 4061.0 |
|  | 60 | 8046.0 | 6622.7 | 4932.8 | 4002.7 | 3449.1 |
|  | 50 | 6697.0 | 5486.4 | 4070.0 | 3289.4 | 2820.8 |
| xii | 100 | 13437.6 | 14171.0 | 12448.2 | 10016.9 | 8458.4 |
|  | 90 | 13477.1 | 13817.1 | 11570.5 | 9220.7 | 7766.1 |
|  | 80 | 13481.9 | 13246.9 | 10394.6 | 8198.5 | 6894.8 |
|  | 70 | 13139.6 | 12242.9 | 9202.4 | 7220.6 | 6067.3 |
|  | 60 | 12271.4 | 10744.5 | 7790.4 | 6093.9 | 5103.9 |
|  | 50 | 10873.1 | 9123.3 | 6542.7 | 5113.0 | 4278.5 |
| xiii | 100 | 13237.5 | 14269.3 | 13615.0 | 11130.2 | 9358.0 |
|  | 90 | 13224.5 | 14099.2 | 12746.4 | 10218.8 | 8569.3 |
|  | 80 | 13271.4 | 13791.1 | 11569.7 | 9121.8 | 7625.5 |
|  | 70 | 13329.0 | 13155.4 | 10322.7 | 8055.9 | 6718.2 |
|  | 60 | 13113.9 | 12186.3 | 9021.3 | 7002.2 | 5827.4 |
|  | 50 | 12035.0 | 10524.6 | 7554.0 | 5852.4 | 4867.5 |

Note: HU values greater than 12,500 have diminished accuracy due to saturation and lack of transmission signal in projection data.

**Supplemental Table 3.** HUs for standard commercial phantom materials plugs. Data plotted in Figures 4, 5, and 6.

| **Material** | **70 kVp** | **80 kVp** | **100 kVp** | **120 kVp** | **140 kVp** |
| --- | --- | --- | --- | --- | --- |
| I | -653.2 | -658.8 | -661.1 | -664.7 | -665.8 |
| II | -489.4 | -492.1 | -502.4 | -502.4 | -502.5 |
| III | -108.7 | -103.5 | -93.9 | -86.6 | -80.4 |
| IV | -53.5 | -50.6 | -45.5 | -44.6 | -41.1 |
| V | 13.6 | 10.8 | 6.0 | 3.9 | 3.7 |
| VI | -2.1 | 9.5 | 20.3 | 24.4 | 30.7 |
| VII | 84.7 | 86.1 | 84.2 | 80.5 | 81.9 |
| VIII | 364.4 | 321.3 | 262.3 | 226.5 | 207.8 |
| IX | 367.9 | 321.8 | 262.3 | 227.5 | 210.4 |
| X | 166.7 | 177.1 | 194.2 | 204.8 | 210.6 |
| XI | 655.0 | 586.6 | 497.0 | 444.5 | 413.3 |
| XII | 1215.6 | 1080.6 | 903.1 | 798.0 | 735.2 |
| XIII | 1839.0 | 1630.1 | 1360.3 | 1195.8 | 1095.9 |
| XIV | 4002.3 | 3584.4 | 3089.5 | 2816.0 | 2645.9 |
| XV | 16623.8 | 14680.3 | 11604.9 | 9714.9 | 8576.3 |
| XVI | 18741.7 | 19741.3 | 20456.6 | 19010.3 | 17193.5 |

Note: HU values greater than 12,500 have diminished accuracy due to saturation and lack of transmission signal in projection data.
